# Necrotizing Autoimmune Myopathy With HMGCR Antibodies: An Uncommon Presentation of Autoimmune Myositis in a Middle-Aged Woman Without Known Malignancy, Connective Tissue Disorder, or Statin Therapy

**DOI:** 10.7759/cureus.97009

**Published:** 2025-11-16

**Authors:** Manoj Kumar Mahadevaswamy Susheela, Rekha Kethohalli Shivamurthy, Sathia Narayanan Mannath, Stephen Proctor, Cornelius James Fernandez

**Affiliations:** 1 Diabetes and Endocrinology, United Lincolnshire Teaching Hospitals NHS Trust, Boston, GBR; 2 Internal Medicine, United Lincolnshire Teaching Hospitals NHS Trust, Boston, GBR; 3 Nephrology, University Hospitals of Derby and Burton NHS Foundation Trust, Derby, GBR; 4 Endocrinology, United Lincolnshire Teaching Hospitals NHS Trust, Lincoln, GBR

**Keywords:** autoimmune necrotizing myositis, muscle biopsy, musculoskeletal mri, myositis-specific antibodies, systemic steroid therapy

## Abstract

We report an uncommon presentation of necrotizing autoimmune myopathy (NAM) associated with anti-HMGCR antibodies in a middle-aged woman who lacked typical risk factors, including statin use, malignancy, or connective tissue disease. The patient presented with muscle weakness, markedly elevated creatine kinase levels, and muscle biopsy findings consistent with necrosis. Diagnosis of NAM was confirmed through the detection of anti-HMGCR antibodies. Despite the initiation of corticosteroid therapy, the condition progressed rapidly, highlighting the aggressive nature of this form of autoimmune myositis. This case underscores the importance of considering NAM in the differential diagnosis of idiopathic myopathy, even in the absence of common predisposing factors, and emphasizes the need for early detection and individualized treatment strategies.

## Introduction

Autoimmune myositis (AIM), previously known as idiopathic inflammatory myopathies (IIM), comprises a group of inflammatory disorders affecting skeletal muscle. AIM includes pure polymyositis (PM), pure or classic dermatomyositis (DM), immune-mediated necrotizing myopathy or necrotizing autoimmune myopathy (NAM), overlap myositis (OM), and sporadic inclusion body myositis (IBM). Most AIM subgroups have a female predominance (2:1), except IBM, which is two to three times more common in males. While DM can affect both children (juvenile DM or JDM) and adults, other AIM subgroups are typically seen in middle-aged adults. IBM follows a chronic progressive course, whereas other forms of AIM usually present with an acute or subacute onset [1]. Although muscle weakness is the primary symptom, AIM can also involve other organs, including the skin, lungs, and heart, making diagnosis and management complex [2]. Because it is rare and shares clinical features with other muscle disorders, diagnosing AIM can be challenging. However, early recognition is crucial for improving outcomes.

NAM is a subtype of IIM characterized by severe, rapidly progressive proximal muscle weakness and markedly elevated creatine kinase (CK) levels. Anti-HMGCR antibodies are strongly associated with NAM and may occur with or without prior statin exposure. This case report describes a patient with NAM who lacked typical risk factors yet tested positive for HMGCR antibodies, presented with a severe form of the disease, and required immunotherapy. Through this case, we aim to highlight the importance of recognizing AIM, discuss its clinical presentation and diagnostic workup, and provide insights into management strategies.

## Case presentation

**Figure 1:**
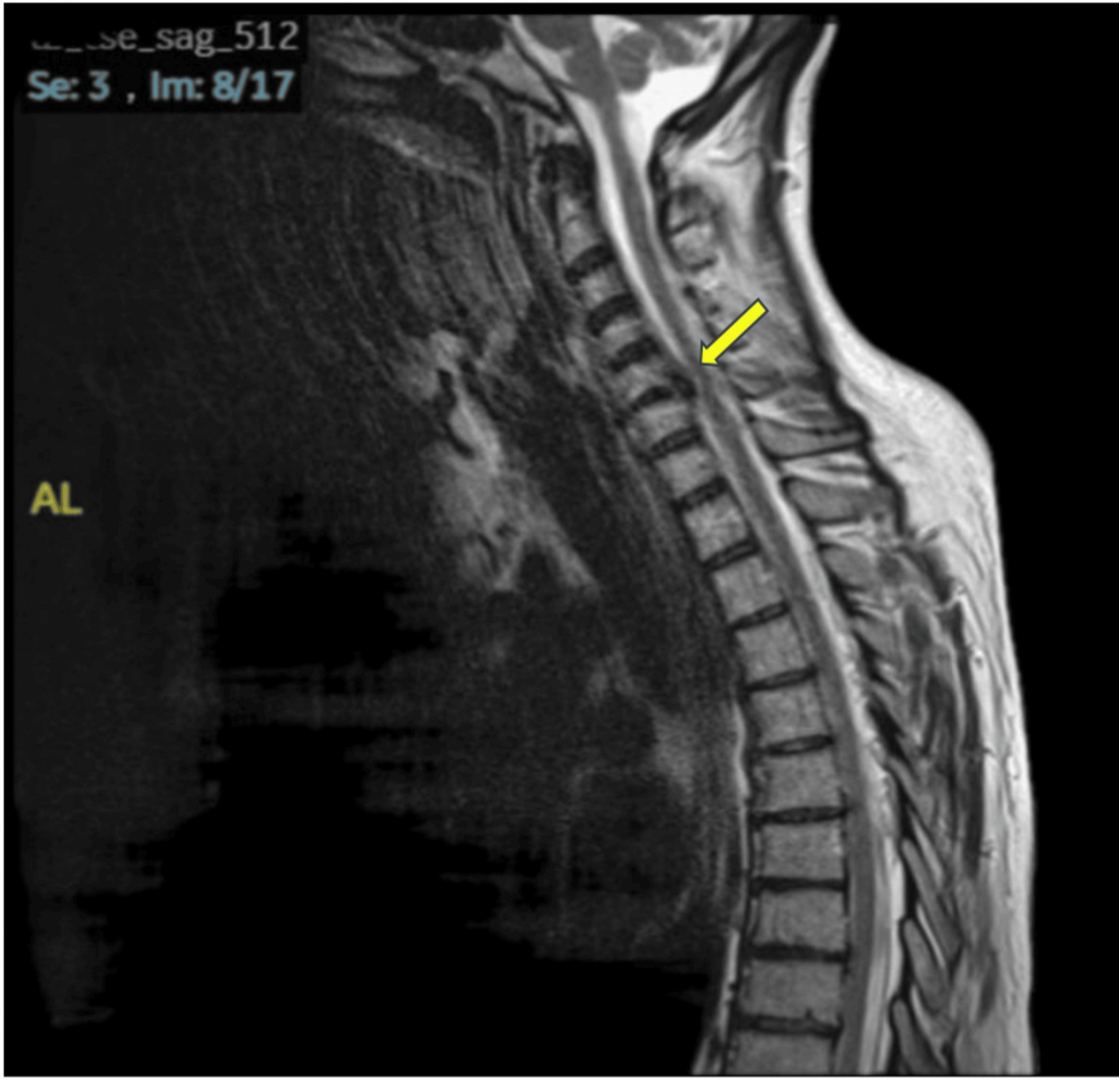
MRI T1 sagittal image of the spine showing a prominent disc bulge at C5/C6 The patient underwent decompression and spinal fusion surgery, but there was no improvement in weakness.

A 46-year-old woman with a history of depression was admitted to our hospital with neck pain and upper limb weakness lasting three months. She underwent a whole-spine MRI, which revealed a prominent disc bulge at C5/C6 compressing the right exiting nerve root. She subsequently underwent decompression surgery with spinal fusion three days later; however, there was no improvement in her upper limb weakness (Figure 1).

However, her weakness persisted, and she was readmitted 10 days later. She reported progressively worsening proximal muscle weakness, which initially manifested as difficulty lifting her head off the bed and subsequently progressed to an inability to raise her upper and lower limbs. She required support to stand, was unable to bear weight, experienced recurrent disabling falls, and developed dysphagia. Eight months prior, she had been treated with a moderately potent topical steroid cream for an erythematous maculopapular rash around her ears, neck, and upper thorax.

She denied muscle or joint pain, Raynaud’s phenomenon, weight loss, or other systemic symptoms and was not taking any medications. There was no relevant family history.

On examination, her BMI was 36 kg/m². There was no obvious muscle wasting. Proximal muscle strength (notably neck flexion, shoulder abduction, and hip flexion) in both upper and lower limbs was 1/5, while distal strength was 4/5. The Manual Muscle Testing 8 (MMT-8) score was less than 60 [3]. Muscle tone, reflexes, sensation, and coordination were all normal.

**Figure 2:**
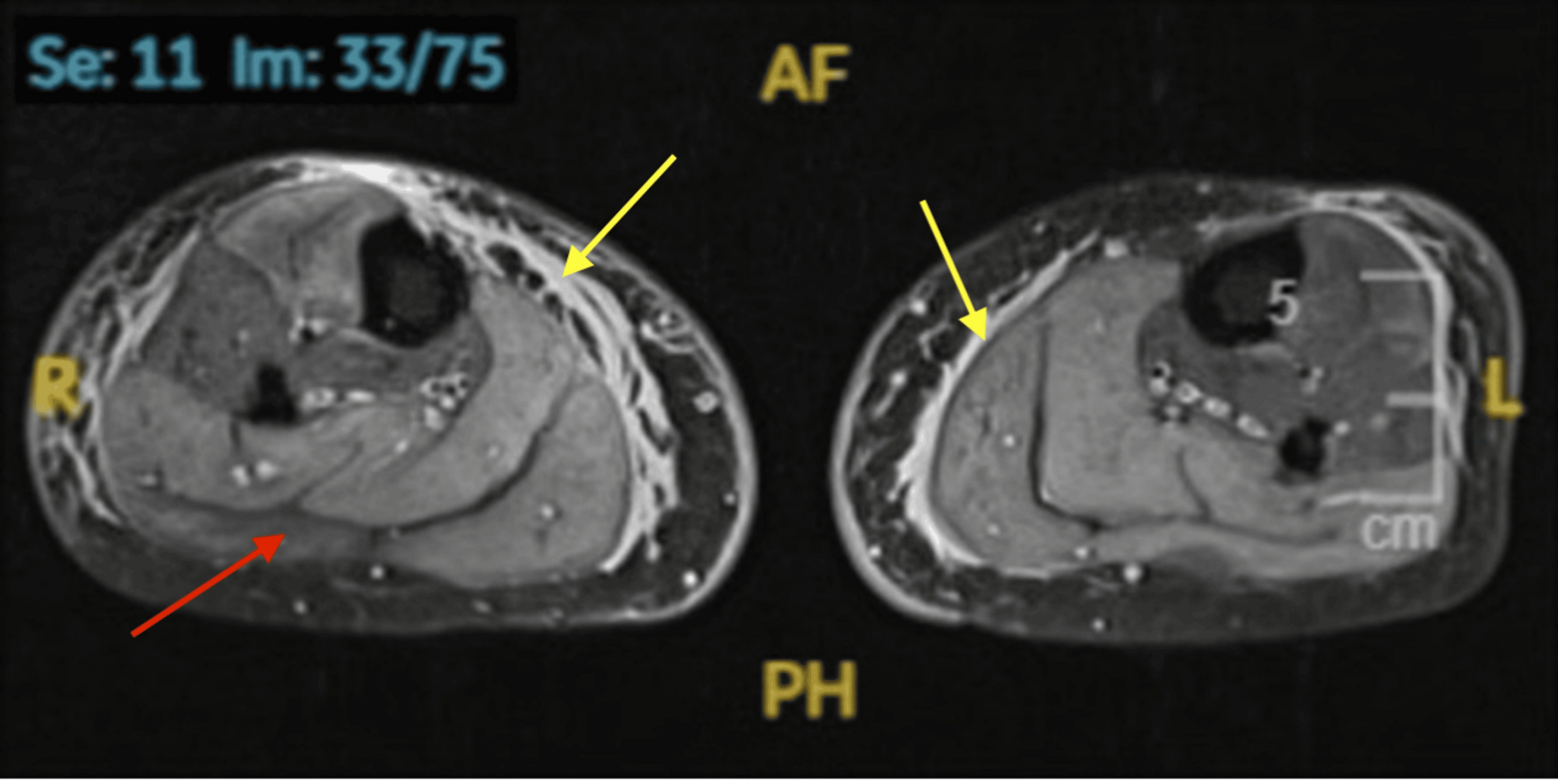
MRI cross-sectional STIR sequence images of the right and left thighs demonstrating generalized bilateral symmetrical muscle edema, suggestive of PM The yellow arrow indicates subcutaneous tissue edema, and the red arrow indicates intermuscular edema. PM, polymyositis; STIR, short tau inversion recovery

Laboratory investigations revealed elevated transaminases and a markedly elevated CK of 7,500 U/L (Table 1). MRI was highly suggestive of PM, demonstrating bilateral symmetrical muscle edema (Figure 2). The serum myositis panel was weakly positive for anti-PM Scl-75 antibody, and antinuclear antibody testing was negative (Table 2). As there was no clinical evidence of malignancy, tumor markers and a CT scan of the chest, abdomen, and pelvis were performed; these were unremarkable except for incidental bilateral benign adrenal adenomas.

**Table 1:** Blood test results showing elevated creatine kinase levels ALT, alanine aminotransferase; CK, creatine kinase; GFR, glomerular filtration rate; HDL, high-density lipoprotein; LDL, low-density lipoprotein

Test	Reference range	Result
Sodium	133-146 mmol/L	142
Potassium	3.5-5.3 mmol/L	3.9
Urea	2.5-5.3 mmol/L	3.3
Creatinine	45-84 µmol/L	30
GFR	>90 mL/min/1.73 m²	>90
Total cholesterol	<5 mmol/L	4.5
Triglycerides	<2 mmol/L	1.9
LDL cholesterol	<3 mmol/L	2.8
HDL cholesterol	>1 mmol/L	0.8
ALT	0-339 U/L	15
Adjusted calcium	2.20-2.60 mmol/L	2.39
CK	25-200 U/L	7500
CRP	0-5 mg/L	4
Hemoglobin	117-149 g/L	139
White blood cells	4.3-11.2 × 10⁹/L	5.9
Platelets	150-400 × 10⁹/L	251
Phosphate	0.8-1.50 mmol/L	0.94

**Table 2:** Autoimmune antibody screening blood tests ANA, antinuclear antibody; ANCA, anti-neutrophil cytoplasmic antibody; Anti-dsDNA Ab, anti-double-stranded DNA antibody; Anti-EJ Ab, anti-glycyl tRNA synthetase antibody; Anti-HMGCR Ab, anti-3-hydroxy-3-methylglutaryl-coenzyme A reductase antibody; Anti-Jo-1 Ab, anti-histidyl tRNA synthetase antibody; Anti-MDA-5 Ab, anti-melanoma differentiation-associated gene 5 antibody; Anti-Mi-2 Ab, anti-Mi-2 antibody; Anti-NXP-2 Ab, anti-nuclear matrix protein 2 antibody; Anti-OJ Ab, anti-isoleucyl tRNA synthetase antibody; Anti-PM/Scl 75 Ab, anti-polymyositis/scleroderma 75 kDa antibody; Anti-PL-7 Ab, anti-threonyl tRNA synthetase antibody; Anti-SAE Ab, anti-small ubiquitin-like modifier activating enzyme antibody; Anti-Scl Ab, anti-scleroderma (topoisomerase I) antibody; Anti-SRP Ab, anti-signal recognition particle antibody

Antibody test	Result
Anti-Jo Ab	Negative
Anti-Mi-2 Ab	Negative
Anti-PL-7 Ab	Negative
Anti-OJ Ab	Negative
Anti-EJ Ab	Negative
Anti-NXP-2 Ab	Negative
Anti-HMGCR Ab	Positive
Anti-MDA-5 Ab	Negative
Anti-SAE Ab	Negative
Anti-SRP Ab	Negative
ANA	Negative
ANCA	Negative
Anti-Scl Ab	Negative
Anti-dsDNA Ab	Negative
Anti-PM/Scl 75 Ab	Weakly positive

The patient was initially treated with 60 mg of oral prednisolone and methotrexate (MTX). Although her CK levels decreased from 7,000 to 1,000 U/L over the following weeks, there was minimal improvement in muscle strength, with MMT-8 remaining below 60. Consequently, she received a three-day course of IV methylprednisolone. Electromyography (EMG) findings were supportive of myopathy.

Approximately three months later, a muscle biopsy revealed necrotizing myopathy on a background of chronic myopathy, with mild inflammatory infiltrates, major histocompatibility complex (MHC) class I upregulation, and perifascicular atrophy, suggestive of a possible diagnosis of DM. In view of the necrosis observed on biopsy, anti-HMGCR antibodies were tested and returned positive, confirming a diagnosis of NAM.

The patient underwent seven cycles of therapeutic plasma exchange, followed by five consecutive days of IV immunoglobulin (IVIG) at a tertiary care center. Following this treatment, her proximal and neck muscle strength improved to 3-4/5, with MMT-8 increasing to 100.

**Figure 3:**
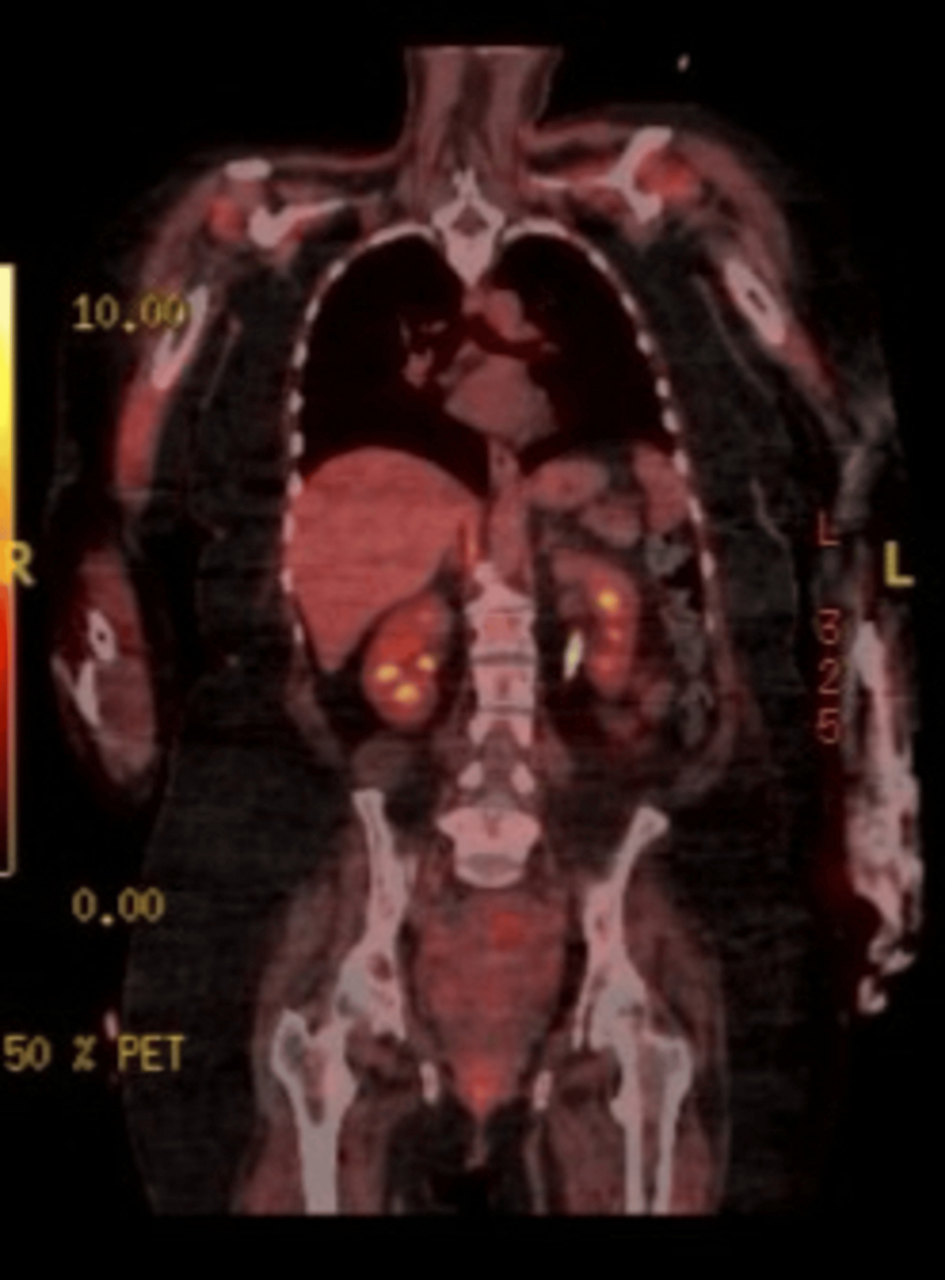
PET scan showing no evidence of malignancy

Given that both DM and NAM are associated with malignancy, a PET scan was performed. The PET scan showed no evidence of malignancy; however, caution was advised, as prior high-dose steroid therapy could reduce PET sensitivity for detecting inflammatory disorders (Figure 3).

**Figure 4:**
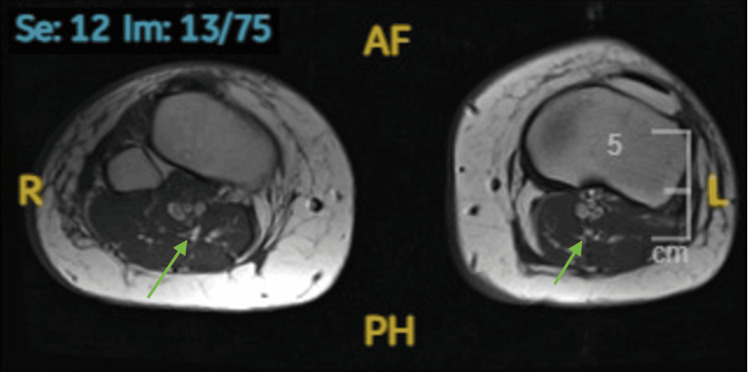
Repeat MRI STIR sequence of the lower limbs showing generalized marked atrophy and edema of muscle groups The green arrow indicates generalized muscle atrophy involving all muscle components, including the soleus, gastrocnemius, and anterior tibial muscles.

A repeat MRI of the lower limbs demonstrated marked atrophy and edema of bilateral lower limb muscle groups (Figure 4). Despite this, approximately 60% of the thigh muscles were preserved. Currently, the patient is undergoing physiotherapy and has achieved functional improvements: she can lift her arms above her head, raise her neck off the pillow, sit unsupported at the edge of the bed with improved balance, and perform sit-to-stand movements with assistance, bearing weight using a Zimmer frame. Her dysphagia has also improved with ongoing speech and language therapy (SALT).

Her steroid dose is being tapered by 5 mg every four weeks until cessation, and MTX is continued at 20 mg per week. She is scheduled to start rituximab (RTX), 1 g administered two weeks apart, with repeat cycles planned every six months.

## Discussion

AIM is a rare group of diseases, with an annual incidence of 0.1-1.0 per 100,000 person-years and a prevalence of 0.55-6.0 per 100,000 person-years [4]. Recent studies, however, suggest that the incidence of AIM has increased to 1.76 per 100,000 person-years, with higher rates in females than males (2.52 vs. 1.0 per 100,000 person-years, respectively) [5]. OM accounts for nearly 50% of AIM cases, while pure DM represents 30-35%, and NAM constitutes 10-15% of cases. The incidence of PM, now considered a diagnosis of exclusion, has declined significantly and represents only about 5% of AIM cases [1].

Clinical features

The major symptoms of AIM include acute (within days) or subacute (within weeks) weakness of the proximal muscles of the arms and legs, often involving the neck flexors and frequently associated with muscle pain. AIM is typically accompanied by a 10- to 50-fold elevation in CK levels, except in sporadic IBM, where CK elevation is usually less than 15-fold [6]. Patients with pure DM often exhibit characteristic cutaneous signs, including Gottron’s papules (erythematous papules or plaques over the dorsal aspect of the fingers and hands), heliotrope rash (periorbital edema and erythema), V-sign (erythema over the upper anterior chest), shawl sign (erythema over the posterior neck), periungual erythema with telangiectasia, Samitz’s sign (ragged cuticles), and Holster sign (erythema over the lateral hip) [6,7]. These rashes are not exclusive to DM and may also occur in OM patients. Case reports indicate that NAM patients can rarely present with rashes mimicking DM; however, rashes are generally absent in NAM [8].

Another feature common to both DM and OM is “mechanic’s hands,” characterized by thickened, cracked skin over the ventral and dorsal surfaces of the fingers. Several variants of DM have been described. Amyopathic DM (ADM) presents with skin manifestations without muscle involvement, whereas adermatopathic DM (DM sine dermatitis) involves muscle without skin lesions and often overlaps with OM. Juvenile DM typically presents in children with fever, rash, and frequently cutaneous calcinosis (Table 3) [6].

**Table 3:** Myositis with rash: differentiating points between pure DM vs. OM with DM rash CK, creatine kinase; DM, dermatomyositis; ILD, interstitial lung disease; OM, overlap myositis Source: [8]

Feature	Pure DM	OM with DM features
Onset	Acute or subacute	Acute or subacute
Rash	Present	Present
Nailfold	Abnormal nailfold capillaries	Abnormal nailfold capillaries
CK levels	Normal to 10- to 15-fold	10- to 15-fold
First feature	Rash; later proximal weakness	Proximal weakness; later rash
Characteristics of the rash	Classic and extensive; chronic/refractory to therapy	Isolated heliotrope rash/Gottron’s papules; discrete but transient rash
Overlap features	Not significant	Significant
Dysphagia	Significant (oropharyngeal)	Not significant
Autoantibodies	DM-specific autoantibodies	OM-specific autoantibodies
Chance of cancer	50%	<5%
Therapy response	Good, except for cancer/ILD	Good, except for cancer/ILD

OM is characterized by the coexistence of myositis and features of connective tissue disease (CTD), including Raynaud’s phenomenon, arthritis, mechanic’s hands, interstitial lung disease (ILD), trigeminal neuropathy, lower esophageal dysmotility, systemic sclerosis (SSc), systemic lupus erythematosus (SLE), and Sjögren syndrome (SS). OM represents a heterogeneous subgroup encompassing several conditions, the most common being anti-synthetase syndrome (ASS), which is associated with anti-transfer RNA synthetase autoantibodies (ASAs) [1]. In addition to the eight ASAs, five other autoantibodies previously classified as myositis-associated autoantibodies (MAAs) are linked to OM [6].

NAM generally exhibits a more severe and rapidly progressive clinical course compared to DM and PM. Approximately 10-20% of NAM patients are positive for SRP antibodies, whereas 60% have HMGCR antibodies. NAM patients with SRP antibodies typically show poor response to immunosuppressive therapy and may develop irreversible disease. In contrast, those with HMGCR antibodies often respond well to immunosuppressive therapy, with reversible muscle necrosis. Among HMGCR-positive patients, 30-60% report prior statin exposure. NAM cases with SRP antibodies or statin-exposed HMGCR-positive NAM are generally not associated with malignancy, whereas statin-naïve HMGCR-positive NAM and seronegative NAM may be linked to cancer [6]. Additionally, NAM can coexist with CTDs, though nearly half of the cases remain idiopathic [9].

Autoantibodies

Approximately 60-80% of AIM patients have detectable autoantibodies [10]. These autoantibodies are traditionally divided into two groups: MAAs and myositis-specific autoantibodies (MSAs). They are useful for predicting additional complications and assessing response to treatment. MAAs are commonly detected in patients with CTD overlap, such as in OM, whereas MSAs are specific to other forms of AIM, including ASS, which is associated with ASAs [11]. Although MAAs such as Ku, U1RNP, PM/Scl, SSA/Ro52/Ro60, and SS-B/La are detectable in nearly 50% of AIM patients, they are not disease-specific. In contrast, MSAs have a specificity exceeding 90% for diagnosing AIM [12]. However, their sensitivity is only 60-70% [13], indicating that a negative result does not rule out the diagnosis (Table 4).

**Table 4:** Clinical spectrum associated with various autoantibodies of AIM AIM, autoimmune myositis; ASA, anti-synthetase antibody; cN1A, cytosolic 5’-nucleotidase 1A; CTD, connective tissue disease; DM, dermatomyositis; EJ, anti-glycyl tRNA synthetase antibody; Ha, anti-tyrosyl tRNA synthetase antibody; HMGCR, 3-hydroxy-3-methylglutaryl-coenzyme A reductase antibody; IBM, inclusion body myositis; ILD, interstitial lung disease; JDM, juvenile dermatomyositis; Jo-1, anti-histidyl tRNA synthetase antibody; KS, anti-asparaginyl tRNA synthetase antibody; Ku, anti-Ku antibody; MDA5, melanoma differentiation-associated gene 5; Mi-2, Mi-2 antibody; NAM, necrotizing autoimmune myopathy; NXP-2, nuclear matrix protein 2; OJ, anti-isoleucyl tRNA synthetase antibody; PL-7, anti-threonyl tRNA synthetase antibody; PL-12, anti-alanyl tRNA synthetase antibody; PM/Scl, polymyositis/scleroderma antibody; Ro52/Ro60, Sjögren’s-syndrome-related antigen A; RTX, rituximab; SAE, small ubiquitin-like modifier activating enzyme; SLE, systemic lupus erythematosus; SS, Sjögren’s syndrome; SS-B/La, Sjögren’s-syndrome-related antigen B; SRP, signal recognition particle antibody; SSc, systemic sclerosis; TIF-1, transcription intermediary factor 1; U1RNP, U1 ribonucleoprotein; Zo, anti-phenylalanyl tRNA synthetase antibody Source: [11]

AIM subgroup	Autoantibody		Frequency	Clinical feature
DM	MDA5		15-30 of DM	Amyopathic DM, IL
	Mi-2		5-10% of DM	Classical DM; good response
	TIF-1		20% of DM	Cancer 75%; first in JDM
	NXP-2		10-15% of DM	Cancer 37.5%; second in JDM
	SAE		2-8% of DM	Amyopathic DM; dysphagia
OM	tRNA synthetase (30% of AIM)	Jo-1	15-20%	Increased ILD and mortality rate associated with non-Jo-1 ASAs compared to Jo-1. Presence of ASAs predicts good response to ILD with RTX.
		PL-7	3-4%	
		PL-12	3-4%	
		OJ, KS, Zo, EJ, Ha	<2%	
	Ku		20-30% of OM	SSc, SLE, CTD; poor response
	U1RNP		10% of OM	SSc, SLE, CTD; good response
	PM/Scl		8-10% of OM	SSc; severe; poor response; ILD
	SSA/Ro52/Ro60		25% of OM	SS, SLE, SSc, CTD
	SS-B/La		12% of OM	
NAM	SRP		5% of AIM	Severe; irreversible; with ILD
	HMGCR		5-8% of AIM	Statin; reversible; cancer 11%
IBM	cN1A		30% of IBM	Severe; chronic; high mortality

Comorbid conditions

All AIM subgroups, except IBM, are associated with a 2- to 7-fold increased risk of malignancy compared to the general population. The highest incidence is observed in patients with TIF-1, NXP-2, and HMGCR autoantibodies. Cancer risk is greatest within one to three years before and after the diagnosis of AIM. The most common malignancies include lung, breast, and ovarian cancers, as well as lymphoma. Evaluation for malignancy should include CT or MRI of the chest and abdomen, PET scans, tumor markers, with or without specialist referral, and endoscopy. Even if initial results are normal, imaging should be repeated annually for at least three years [6].

ILD, another comorbidity that increases morbidity and mortality in AIM, is associated with tRNA synthetase, U1RNP, PM/Scl, Ku, MDA5, and SRP antibodies. Workup for ILD requires high-resolution CT of the chest and pulmonary function testing [6]. Cardiac involvement in OM, including cardiomyopathy, serositis, pericarditis, or conduction disturbances, should be evaluated with 24-hour ECG monitoring, echocardiography, and cardiac MRI. Similarly, OM-associated oropharyngeal and esophageal dysmotility requires assessment by SALT, video fluoroscopy, or real-time MRI, with supportive interventions such as nasogastric or percutaneous feeding as needed. IBM-associated esophageal dysmotility may require local botulinum toxin injections [6].

Diagnostic workup: MRI, EMG, and muscle biopsy

Muscle MRI allows for assessment of the extent and pattern of inflammation, atrophy, and fatty replacement in skeletal muscle. By identifying the most appropriate muscle for biopsy, MRI can improve the diagnostic yield of muscle biopsy [14]. However, MRI findings are not always specific; for instance, intramuscular edema may also be seen in trauma, myonecrosis, infection, rhabdomyolysis, and noninflammatory myopathies [15]. EMG changes are similarly non-specific and cannot reliably differentiate between AIM subgroups. EMG is therefore recommended primarily when an alternative neurological diagnosis is suspected (Table 5) [16].

**Table 5:** Muscle biopsy features that differentiate various subgroups of AIM AIM, autoimmune myositis; CD8+ T cells, cluster of differentiation 8 positive T cells; DM, dermatomyositis; IBM, inclusion body myositis; MHC, major histocompatibility complex; NAM, necrotizing autoimmune myopathy; OM, overlap myositis; PM, polymyositis Source: [6]

AIM subgroup	Muscle biopsy features
DM	Perimysial inflammation; perifascicular atrophy; perifascicular MHC class I upregulation; complement binding to capillaries and/or sarcolemma; capillary loss
OM	Perifascicular necrosis; perifascicular MHC class I and II upregulation; complement binding to sarcolemma
PM	Endomysial CD8+ T cells; widespread upregulation of MHC class I
NAM	Scattered necrosis of myofibers; focal upregulation of MHC class I in necrotic areas; complement binding to capillaries and/or sarcolemma
IBM	Endomysial CD8+ T cells; widespread upregulation of MHC class I; amyloid deposition; presence of vacuoles and tubulofilaments; mitochondrial impairment

Classification

The European League Against Rheumatism/American College of Rheumatology (EULAR/ACR) criteria (2017) use clinical parameters including age at diagnosis, pattern of weakness, skin manifestations, esophageal dysmotility, laboratory features (Jo-1 autoantibody and muscle enzymes), and muscle biopsy findings. A simple algorithm classifies AIM into JDM, DM, ADM, IBM, and PM, with or without biopsy data. The algorithm provides a score corresponding to the probability of AIM: 50-55% indicates a possible diagnosis, 55-90% probable, and >90% definite [17]. A web-based calculator is also available for ease of use [18]. When combined with biopsy, these criteria have an overall sensitivity of 93% and specificity of 88%, whereas without biopsy, sensitivity decreases to 87% and specificity to 82%.

Several limitations of the EULAR/ACR criteria exist. Subgroups such as OM and NAM are not considered as separate entities, antibodies other than Jo-1 are not included in decision-making, and many newly proposed biopsy features of AIM are excluded [19]. Muscle biopsy is therefore essential for categorizing AIM subgroups and differentiating AIM from non-inflammatory myopathies, with MHC labeling aiding distinction from muscular dystrophies. Muscle biopsy is recommended for all AIM cases except classical DM [20].

Although perifascicular atrophy and MHC class I upregulation, features seen in DM, were observed in the biopsy, the histology was dominated by muscle fiber necrosis with minimal inflammatory infiltrate, characteristic of NAM. The positive anti-HMGCR antibody, absence of DM-specific antibodies, and lack of pathognomonic cutaneous or systemic features support NAM as the predominant process, suggesting NAM with overlap features rather than classic DM.

Treatment

The treatment of all AIM forms is broadly similar, except for IBM. The goals are to eliminate muscle inflammation, restore motor function, and prevent chronic muscle damage and other organ dysfunction.

Nonpharmacological Therapy

A multidisciplinary approach is essential. Physiotherapy should begin early and has been shown to safely improve muscle strength, aerobic conditioning, and quality of life in AIM patients [21]. Dysphagia should be managed with SALT. Adequate sun protection and reduced UV exposure are recommended for dermatological manifestations.

Pharmacological Therapy

Prednisolone is first-line therapy (1 mg/kg/day) for four to 12 weeks, followed by gradual tapering every four weeks depending on clinical response, often maintaining a dose of 5-10 mg/day. Alternate-day high-dose steroids may improve efficacy and reduce long-term side effects [22]. In severe cases, IV methylprednisolone (1000 mg/day for three to five consecutive days) may be used. Long-term steroid therapy requires calcium and vitamin D supplementation, lifestyle measures, and bisphosphonate therapy to prevent osteoporosis.

For moderate to severe refractory AIM or disease flare during steroid tapering, immunosuppressants such as MTX, azathioprine (AZA), or mycophenolate mofetil (MMF) may be added, with choice guided by comorbidities. A recent Cochrane review found no significant difference in efficacy among these agents [23]. MTX requires folic acid supplementation and pre-screening for liver and lung disease. Side effects include leukopenia, gastrointestinal upset, transaminitis, pneumonitis, and teratogenicity. MTX is contraindicated in patients with ILD. Regular monitoring of full blood count, liver function tests, infection risk, and vaccinations is recommended. AZA is suitable for ILD or liver disease and for patients who consume alcohol. Thiopurine S-methyltransferase-deficient patients are at high risk for AZA-induced bone marrow toxicity and require lower starting doses. MMF is reserved for patients intolerant to MTX or AZA. Therapeutic effects of immunosuppressants may take several weeks [23].

IVIG or cyclosporine can be considered for patients refractory to steroids and immunosuppressants. Cyclosporine may be used as adjunct or replacement therapy, particularly in ILD, though it carries risks of hypertension, renal impairment, and malignancy. IVIG has shown efficacy in severe refractory AIM, improving muscle strength and CK levels, with potential side effects including allergy, thrombosis, and hemolysis [24,25].

RTX or cyclophosphamide should be considered if there is no improvement with standard immunosuppression and IVIG. RTX has shown promise in severe, refractory AIM, with observational studies and a recent randomized controlled trial demonstrating significant improvement in chronic recalcitrant cases [26]. In AIM associated with malignancy, tumor treatment should take priority alongside immunosuppression. No effective treatment exists for IBM; IVIG may be tried in selected patients (Table 6) [6].

**Table 6:** Pharmacological agents used in the management of AIM AIM, autoimmune myositis; AZA, azathioprine; CYC, cyclophosphamide; IVIG, IV immunoglobulin; MMF, mycophenolate mofetil; MTX, methotrexate; RTX, rituximab Source: [23,27]

Drug	Dosing/notes
Prednisolone	Start oral prednisolone at 1 mg/kg/day (maximum 80 mg) for four to 12 weeks. Taper every four weeks by 20-25% to a maintenance dose of 5-10 mg/day.
Methylprednisolone	IV methylprednisolone 1,000 mg/day for three to five consecutive days.
MTX	Start oral or subcutaneous MTX with folic acid 10-15 mg/week; titrate up to a target dose of 25 mg/week.
AZA	Start oral AZA 50 mg/day; increase in increments of 25-50 mg every one to two weeks to standard 1.5 mg/kg/day (maximum 2.5 mg/kg/day).
MMF	Start oral MMF 250-500 mg twice daily; increase in increments of 250-500 mg every one to two weeks to 2,000-3,000 mg/day.
IVIG	IVIG 1-2 g/kg divided over five doses. Repeat monthly for three to six months based on response. Total dose or frequency can be reduced if responding.
Cyclosporine	Start oral cyclosporine 50 mg twice daily; increase up to 100-150 mg twice daily.
RTX	Two IV doses of 1 g, two weeks apart. Repeat every six months as needed.
CYC	Start oral CYC 50-75 mg/day; titrate up to 1.5-2 mg/kg/day.

Prognostic factors

Poor prognostic factors in AIM include older age, male sex, non-Caucasian ethnicity, delayed treatment initiation, association with malignancy, dysphagia, and respiratory or cardiac complications [28]. During immunosuppressive therapy, improvement in muscle strength is a more reliable marker of treatment response than a decline in CK levels.

Learning points

In patients presenting with neck pain, neck weakness, and upper limb weakness, AIM should be considered in the differential diagnosis alongside more common causes such as cervical disc disease. The most frequent causes of AIM with rash are DM and OM, although NAM can rarely present with rash. NAM is commonly associated with statin use, malignancy, or CTD; however, nearly half of NAM cases remain idiopathic. Accurate diagnosis of NAM requires histological evidence from muscle biopsy as well as detection of myositis-specific antibodies, such as HMGCR and SRP antibodies. While NAM often proves refractory to steroid monotherapy, early and aggressive immunotherapy can improve outcomes in patients with HMGCR antibodies. In contrast, NAM associated with SRP antibodies typically remains unresponsive to immunosuppressive therapy. Given that NAM with HMGCR antibodies may be linked to malignancy, surveillance imaging should be repeated annually for at least three years, even when initial results are negative.

## Conclusions

Our patient was initially diagnosed with cervical disc disease and underwent neurosurgical intervention for neck pain and upper limb weakness. On subsequent admissions, she presented with lower limb weakness, recurrent falls, and dysphagia. Laboratory evaluation revealed markedly elevated CK, and PM was initially considered, supported by MRI of the thighs and EMG findings. Given her prior history of rash and muscle biopsy findings, a diagnosis of classic DM was entertained. However, the detection of HMGCR antibodies, rapidly progressive proximal weakness, high CK, absence of DM-specific antibodies, and lack of pathognomonic cutaneous or systemic features led to a revised diagnosis of NAM. She failed to respond to oral prednisolone, IV methylprednisolone, and MTX but subsequently showed improvement with therapeutic plasma exchange followed by IVIG. At six months, the patient remained in partial remission, with improving functional mobility (MMT-100) and no evidence of malignancy, though close follow-up is planned due to her elevated malignancy risk.

This case underscores the importance of obtaining a detailed clinical history, performing repeated examinations, ordering appropriate investigations, revising the diagnosis when new evidence emerges, and employing a multidisciplinary approach in the diagnosis and management of rare or atypical presentations of autoimmune myopathies. Clinically, it highlights a case of autoimmune necrotizing myopathy without typical risk factors, presenting with progressive muscle weakness, elevated CK, and dysphagia. The diagnosis was confirmed through autoimmune antibody testing supported by muscle biopsy, and successful management was achieved with plasma exchange and IVIG after a poor response to conventional steroid therapy, illustrating the value of a tailored, multidisciplinary treatment strategy.
